# Non-contact elasticity contrast imaging using photon counting

**DOI:** 10.1117/1.JBO.29.7.076003

**Published:** 2024-07-10

**Authors:** Zipei Zheng, Yong Meng Sua, Shenyu Zhu, Patrick Rehain, Yu-Ping Huang

**Affiliations:** Stevens Institute of Technology, Center for Quantum Science and Engineering, Department of Physics, Hoboken, New Jersey, United States

**Keywords:** optics, elastography, light, lasers, tissue-mimicking phantoms, quantum parametric mode sorting

## Abstract

**Significance:**

Tissues’ biomechanical properties, such as elasticity, are related to tissue health. Optical coherence elastography produces images of tissues based on their elasticity, but its performance is constrained by the laser power used, working distance, and excitation methods.

**Aim:**

We develop a new method to reconstruct the elasticity contrast image over a long working distance, with only low-intensity illumination, and by non-contact acoustic wave excitation.

**Approach:**

We combine single-photon vibrometry and quantum parametric mode sorting (QPMS) to measure the oscillating backscattered signals at a single-photon level and derive the phantoms’ relative elasticity.

**Results:**

We test our system on tissue-mimicking phantoms consisting of contrast sections with different concentrations and thus stiffness. Our results show that as the driving acoustic frequency is swept, the phantoms’ vibrational responses are mapped onto the photon-counting histograms from which their mechanical properties—including elasticity—can be derived. Through lateral and longitudinal laser scanning at a fixed frequency, a contrast image based on samples’ elasticity can be reliably reconstructed upon photon level signals.

**Conclusions:**

We demonstrated the reliability of QPMS-based elasticity contrast imaging of agar phantoms in a long working distance, low-intensity environment. This technique has the potential for in-depth images of real biological tissue and provides a new approach to elastography research and applications.

## 1 Introduction

The biomechanical properties of soft tissue, including elasticity, viscosity, and stiffness, have played a crucial role in medical diagnosis throughout history. One example is palpation, a diagnostic technique involving manual pressure applied to a patient’s skin to detect hardness within the body’s structures, which can be traced back to at least 1550 BC in ancient Egypt.^1^ Analog to palpation, elastography is a non-invasive imaging technique used to visualize tissue elasticity. Modern elastography methods typically involve three steps: inducing tissue deformation or vibration, detecting the amplitude or propagation of the deformation or vibration, and using the collected data to calculate elasticity and reconstruct an image.^2^ Elastography techniques include magnetic resonance elastography, ultrasound elastography, Brillouin microscopy, optical coherence elastography (OCE), and others (see Ref. 2 for a review), each with its specific spatial resolution, penetration depth, and application field. In comparison, magnetic resonance elastography offers a spatial resolution of 1 to 3 mm and can penetrate up to 5 cm when detecting the prostate.^3^ It is also applicable to brain tissues, heart, liver, and breast.^2^ Ultrasound elastography provides better spatial resolution, reaching over 100  μm for detecting lesions in breasts, livers, and corneas. However, it is not suitable for smaller organs. To address this, elastography based on Brillouin microscopy has been developed, enabling the detection of elastic properties of ocular tissues and mechanical properties of cells.^4^ It achieves a spatial resolution of 1.5 and 0.3  μm in the axial and lateral directions, respectively, with a penetration depth of 0.1 to 3.0 mm. However, the elasticity measured by Brillouin microscopy corresponds to the longitudinal modulus and does not have a constant relationship with the commonly used Young’s modulus in major biomedical applications.^5^ To apply Brillouin technology in the biomedical field, further research is needed to establish a better understanding of the correlation between longitudinal modulus and Young’s modulus. Finally, OCE is based on optical coherence tomography and reconstructs images using tissue elasticity, offering a spatial resolution of ∼10  μm, a penetration depth of several millimeters, and a field of view of ∼1  cm. Compared with magnetic resonance elastography and ultrasound elastography, OCE is more sensitive to tissue elasticity and provides higher spatial resolution.^2^^,^^6^

Yet, the applications of OCE face several constraints.^7^^,^^8^ The first is the high laser power needed. This is because OCE measures the interference of a reference beam with a highly attenuated and scattered beam so that high laser intensity is required to achieve adequate visibility. The second is a limited working distance because the light scattering from tissues is random and unidirectional so that the collected signal decays quadratically with the distance. To lift these constraints, we explore a new approach to optical elastography using single-photon counting, which we would like to call single-photon elastography (SPE). Unlike OCE, SPE probes the targets using picosecond pulses, collects the signals using a single-mode fiber, and detects them via quantum parametric mode sorting (QPMS). It thus has the advantages of long working distance (up to meters), very low illuminating light intensity (0.16 mW in this experiment, potentially down to a single-photon level), and non-interferometric (as no reference beam is required) and still has potential to reach large penetration depth (up to 10 OD optical depth).^9^ There are two enabling techniques behind SPE. The first is single-photon vibrometry (SPV), where the dynamic speckle pattern created by an oscillating surface is measured by a single-pixel and single-photon-counting light detection and ranging. Similar to OCE, SPV does not use a complex mathematical model to calculate Young’s modulus.^10^ Instead, it directly measures the target’s mechanical response to applied external forces to obtain its elasticity and other physical properties. The second is QPMS, which maximally rejects the background noises from ambient light or multiscattering^11^ so that the ultra-weak, single-photon level signals can be detected reliably despite much stronger noises. This is the key for the technique to work in an ambient environment, long working distance, and high penetration depth.^12^ Table 1 gives a simple comparison with other reported competitive techniques. As seen, our SPE has significant advantages of much lower laser power, being non-contact, and a flexible working distance of up to meters. Also, with a proper laser wavelength, the penetration depth can reach centimeters, thanks to QPMS’ exceptional performance in time gating and noise rejection.^11^^,^^12^^,^^17^ The spatial resolution is limited by the laser beam size, which is ∼2  mm in this experiment (because we use a collimated beam directly from an optical fiber) but can be reduced to microns, similar to other OCE setups.

**Table 1:** Comparison between SPE and other reported OCE.

Reference	Interferometric?	Average Power	Contact/non-contact	Working distance
Ref. 13	Yes	7 mW	Contact	160 mm
Ref. 14	Yes	>10 mW	Contact	50 mm
Ref. 15	Yes	36 mW	Non-contact	20 mm
Ref. 16	Yes	4.6 mW	Non-contact	42.3 mm
This work	No	0.16 mW	Non-contact	Flexible, up to meters

QPMS works by creating short pulses in signature time-frequency modes of certain amplitude and phase profiles to illuminate targets and then selectively detecting them through a quantum frequency conversion process.^18^^–^^20^ Using a nonlinear waveguide for the conversion whose phase matching bandwidth is slightly narrower than the pulses’ spectral width, only the photons in the signature modes can be efficiently converted but not in the other modes.^17^^,^^21^ As such, the signal photons are detected preferably than the noise, leading to a significant advantage in the measurement’s signal-to-noise ratio.^17^ Thus far, QPMS has been applied to sort time-frequency^17^ and spatiotemporal modes.^22^^,^^23^ QPMS-based 3D imagers have also been demonstrated with exceptional penetration depth, loss tolerance, and noise resilience.^11^^,^^12^^,^^24^ In this work, we apply QPMS for elastography. While our current demonstrations are on 2D imaging only, the same technique can be used to construct 3D elastograms with considerable penetration depth.

Our system utilizes the positive correlation between Young’s modulus and the natural vibration frequency of agar phantoms due to their concentrations.^25^ That is, agar phantoms with higher concentrations exhibit greater hardness and thus higher natural frequencies. When excited by sound waves, two agar samples of different concentrations respond differently. For example, if the sound wave is at a lower frequency (e.g., ∼1  kHz), the agar with a lower concentration has a higher vibration amplitude. On the other hand, for higher-frequency sound waves (e.g., ∼5  kHz), the agar with a higher concentration vibrates with a higher amplitude. By illuminating the vibrating phantoms with picosecond pulses, the reflected photons carry a periodic changing speckle pattern due to their surface roughness, density inhomogeneity, and/or random scattering inside the samples. As a result, the number of backscattered photons collected into a single-mode fiber oscillates too. This is because the outgoing probe beam is in a well-defined spatio-temporal mode. The returning photons are, on the other hand, in a distorted mode $\psi (x^{\prime },y^{\prime },t^{\prime })$ that carries the fingerprint of the oscillating speckle pattern. By coupling them into a single-mode fiber centered at (x,y) with transverse mode $\rho (x^{\prime }-x,y^{\prime }-y)$ and using QPMS to selectively count the photons in a single time-frequency mode $G(t^{\prime }-t)$ (here t is the relative delay of the pump pulses from the signal; see Ref. 17), the counts ϕ(x,y,t) registered by the single-photon detector at time t are approximately

$$\phi (x,y,t)∼{\left|\int \psi (x^{\prime },y^{\prime },t^{\prime })\rho^{*}(x^{\prime }-x,y^{\prime }-y)G^{*}(t^{\prime }-t)dx^{\prime }\;dy^{\prime }\;dt^{\prime }\right|}^{2}.$$ (1)

The speckle oscillation amplitude and frequency can then be derived for each pixel. By laterally scanning the probe laser beam using a micro-electro-mechanical systems (MEMS) mirror, we get oscillating photon counting histograms ϕ(x,y,t) at different lateral positions (x,y), from which the phantoms’ mechanical properties are derived.

There are multiple ways to analyze the histograms and derive the samples’ elasticity. In this study, we simply perform Fourier transformation on the time-domain data to find their vibration amplitudes on the driving frequencies $f_{d}$, as relative to those at off frequencies. Hence, the photon counting ϕ(x,y,t) is transformed to Φ(x,y,f), whereas the peak value $\Phi (x,y,f_{d})$ and the noise level $\Phi_{\text{noise}}$ are picked. We define the peak-to-noise ratio (PNR) for each pixel P(x,y) in units of dB as

$$P(x,y)=10\times \log_{10}\;\Phi (x,y,f_{d})-10\times \log_{10}\;\Phi_{\text{noise}}.$$ (2)

In this way, an elastogram can be formed. Thanks to QPMS’ excellent performance in detecting weak signal photons while rejecting much stronger background photons, our system works well under ambient, bright-light conditions, and only photon-level signals are needed for each pixel. To construct the 3D elastogram, we will need to scan the depth of the samples by varying the relative time delay of the probe and pump pulses. This is a technique that we have demonstrated using QPMS with millimeter resolution and centimeter penetration depth^12^^,^^17^ and will be employed in our next generation system. With the above advantages, our SPE adds valuable capabilities to elastography and other remote sensing techniques in terms of robustness, sensitivity, and applicability.

## 2 Method

### 2.1 Material and Preparation Procedure

We use agar as the testing material in this experiment because its elasticity is directly related to its concentration, with good consistency and easy malleability.^26^ Previous studies have also demonstrated that agar-based phantoms, which combine agar with other materials, can effectively mimic brain and muscle tissue. These phantoms exhibit similar acoustical, thermal, and magnetic properties,^27^ showcasing their potential for future research.

To prepare the agar samples, 0.4 g of agar powder (brand: Lenith) is first weighted using an electric scale and a clean glass beaker. Then, boiling tap water is added to the beaker with a pipette to 10 g. The mixture is swirled until all agar powder clumps disappear. Next, the beaker is placed in a microwave oven and heated for 30 s until a clear solution is obtained. This 2% W/W solution is then poured into a silicone letter mold (letter height ∼3.81  cm) and cooled for 10 min in a refrigerator at ∼−4°C. Then, the 2% W/W agar letter is taken out from the mold and put in the bottom of a square silicone mold with a side length of 5 cm. Next, a 5% W/W solution is obtained similarly by adding 2.5 g powder in the beaker and filling the water to 50 g. This 5% W/W solution is then poured into the square mold until the 2% W/W letter is submerged. The mold with a solution and solid letter is put in the same refrigerator and cooled down for 15 min. Finally, a 5% W/W agar block with a 2% W/W letter embedded in the center is obtained, with one side of the letter exposed to air. In our experiment, two agar blocks, each with the letter “T” and “S,” are prepared; see Figs. 5(a) and 5(b). According to Refs. 25 and 26, Young’s modulus is ∼120  kPa for the 2% W/W agar and 700 kPa for the 5% W/W agar. Note that the true values for our samples may deviate because of the tap water we used, the water evaporation loss during the heating processing, and some small air bubbles residing in the sample.

### 2.2 Experiment Setup

**Fig. 1:**
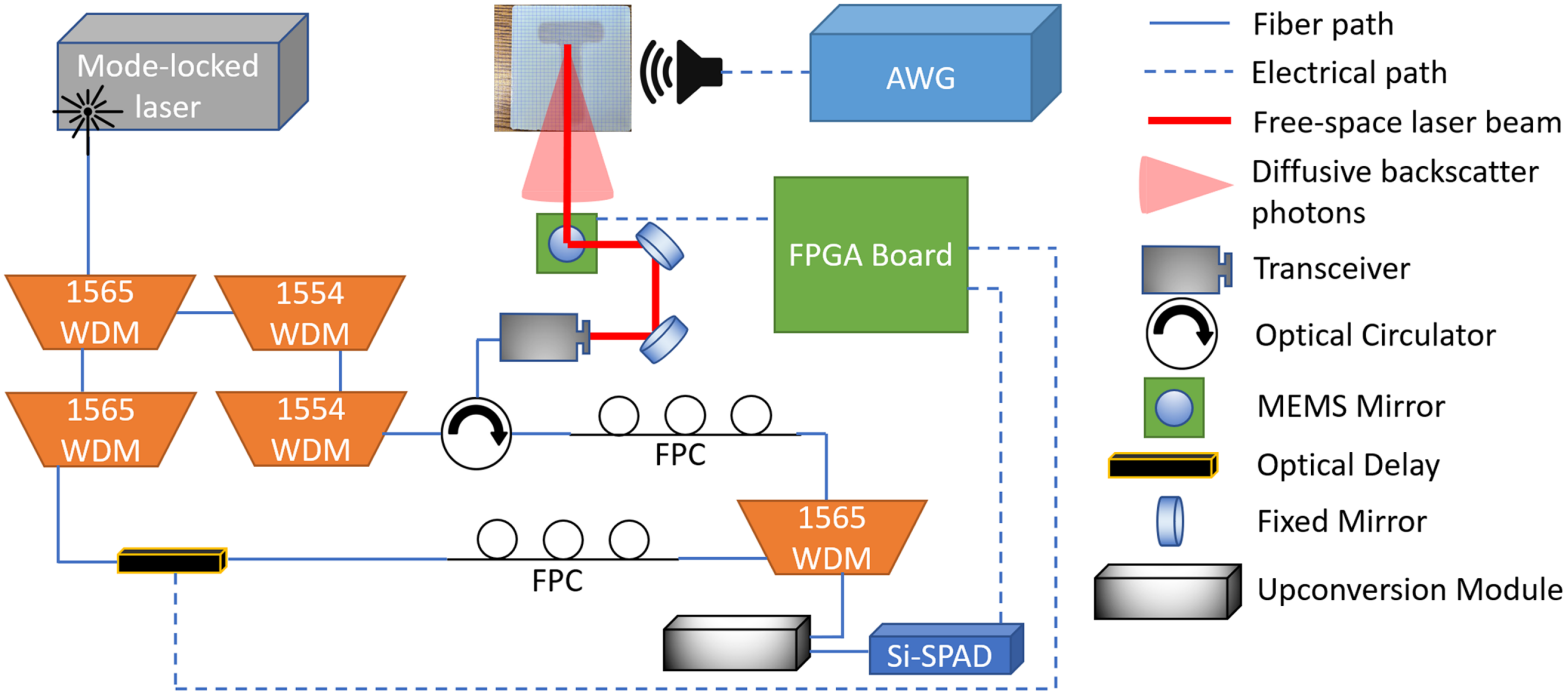
Sketch of the experiment setup. WDM, wavelength division multiplexer; AWG, arbitrary waveform generator; FPC, fiber polarization controller; Si-SPAD, silicon-single-photon avalanche diode.

The experiment setup, as illustrated in Fig. 1, involves the use of a femtosecond mode-locked laser (Calmar FPL-04CFF, California, United States; repetition rate: 50 MHz, center wavelength: 1560 nm) from which two pulse trains are generated using a pair of cascaded wavelength division multiplexers (WDMs) each with a bandwidth of 200 GHz. The laser pulses centered at 1565.5 and 1554.1 nm serve as the pump and probe, respectively. The probe pulse train is transmitted through a fiber-to-free space collimator, which outgoes in the form of a Gaussian beam (beam diameter: 2.2 mm). This beam is reflected by a MEMS mirror and then steered toward the target that is placed 90 cm away. Backscattered signal photons from the target are received in the collimator and separated by an optical fiber circulator with a minimum isolation ratio of 55 dB. The received photons are then recombined with the pump using another WDM and coupled into a mode-selective upconversion module (HC Photonics Corporation, Hsinchu, Taiwan; typical internal conversion efficiency of the waveguide is $85\%/W/{\mathrm{cm}}^{2}$), whose output is sent to a silicon-single-photon avalanche diode (Si-SPAD, Excelitas SPCM-AQRH-12-FC, Massachusetts, United States; detection efficiency ∼70% at 780 nm). An optical delay line (ODL, General Photonics, California, United States; MDL–002) allows for temporal movement of the pump pulse, and when the pump pulse and backscattered signal pulse overlap, the resulting combined photons undergo effective upconversion, where the measured pulse width is 9 ps. The final photon counts are thus related to the convolution of the pump pulses with the signal pulse envelope, enabling us to obtain results from the target at different depths. The Si-SPAD output is sampled by a field-programmable gate array (FPGA) periodically with a preset dwell time and sampling rate. The FPGA is connected to a laptop via an ethernet cable; hence, we are able to analyze the time series of photon counting on a computer by Matlab.

In typical OCE measurements, an external force is applied to the samples to induce displacement, as is also the case with our system. There are two categories of force loading mechanisms: quasi-static compression and dynamic excitation. Within these categories, various methods are used to apply external force, including acoustic radiation force (ARF), air-puff pulse, laser pulse, needle probe, and piezoelectric transducer (PZT).^2^ While the needle probe and PZT actuator methods can generate strong and stable deformations, they are not suitable for measuring vulnerable tissues such as the cornea due to their requirement for physical contact with the sample. On the other hand, ARF, air-puff pulse, and laser pulse can dynamically induce vibration from a distance. However, air-puff and laser pulses only excite the surface of the sample, resulting in limited depth of elasticity detection, making them suitable only for samples located on the body surface.^2^ By contrast, ARF proves to be a better choice as it can remotely excite both the surface and the interior of samples. Consequently, ARF excitation provides the necessary conditions for detecting the elasticity of both superficial and internal tissues. Hence, in this experiment, we decide to use ARF to drive the system.

**Fig. 2:**
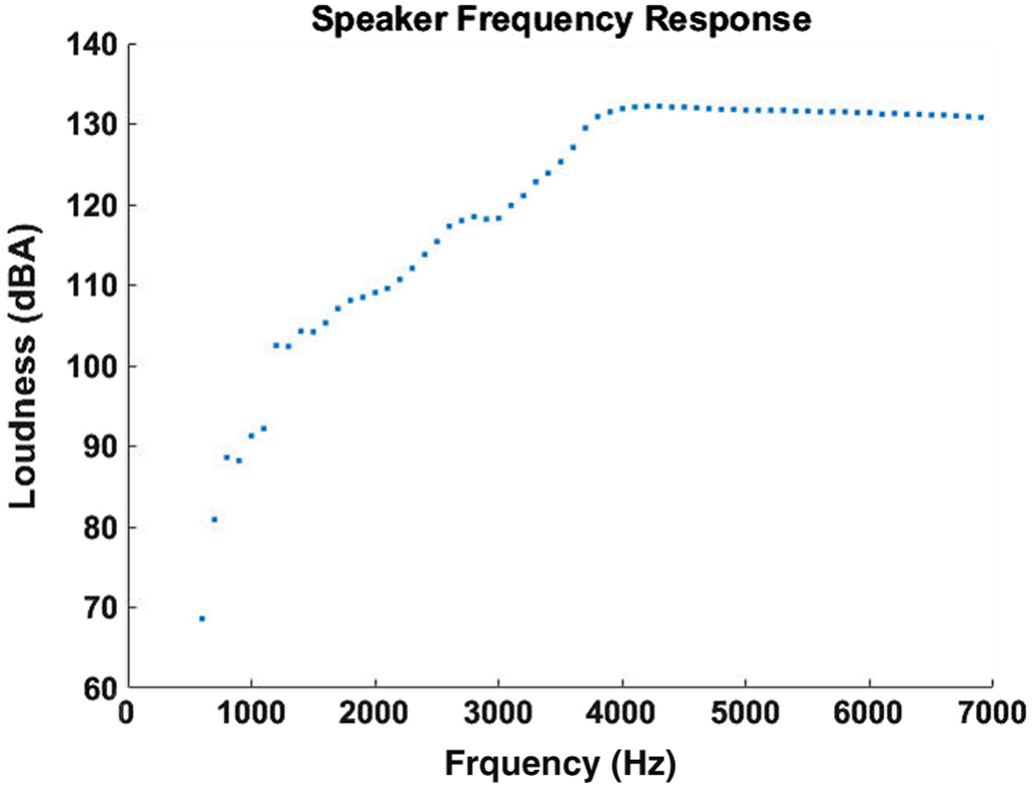
Frequency response of the speaker, measured by a decibel meter 5 mm away.

A piezoelectric speaker (E-outstanding KS-3840A) is utilized in this experiment to produce sound waves, which is connected to an arbitrary waveform generator (AWG, RIGOL DG 4202, Suzhou, China). The speaker is positioned to the left, 5 mm away from the agar phantom without any physical contact, and it faces the agar sample. The AWG generates a sine wave signal with 20 V amplitude. However, due to the non-uniform frequency response of the speaker, the loudness of the sound wave is not constant over the frequency range between 600 and 6900 Hz. Figure 2 plots the speaker loudness measured by a decibel meter (BAFX 3370) as a function of the applied frequency.

### 2.3 Image Reconstruction

**Fig. 3:**
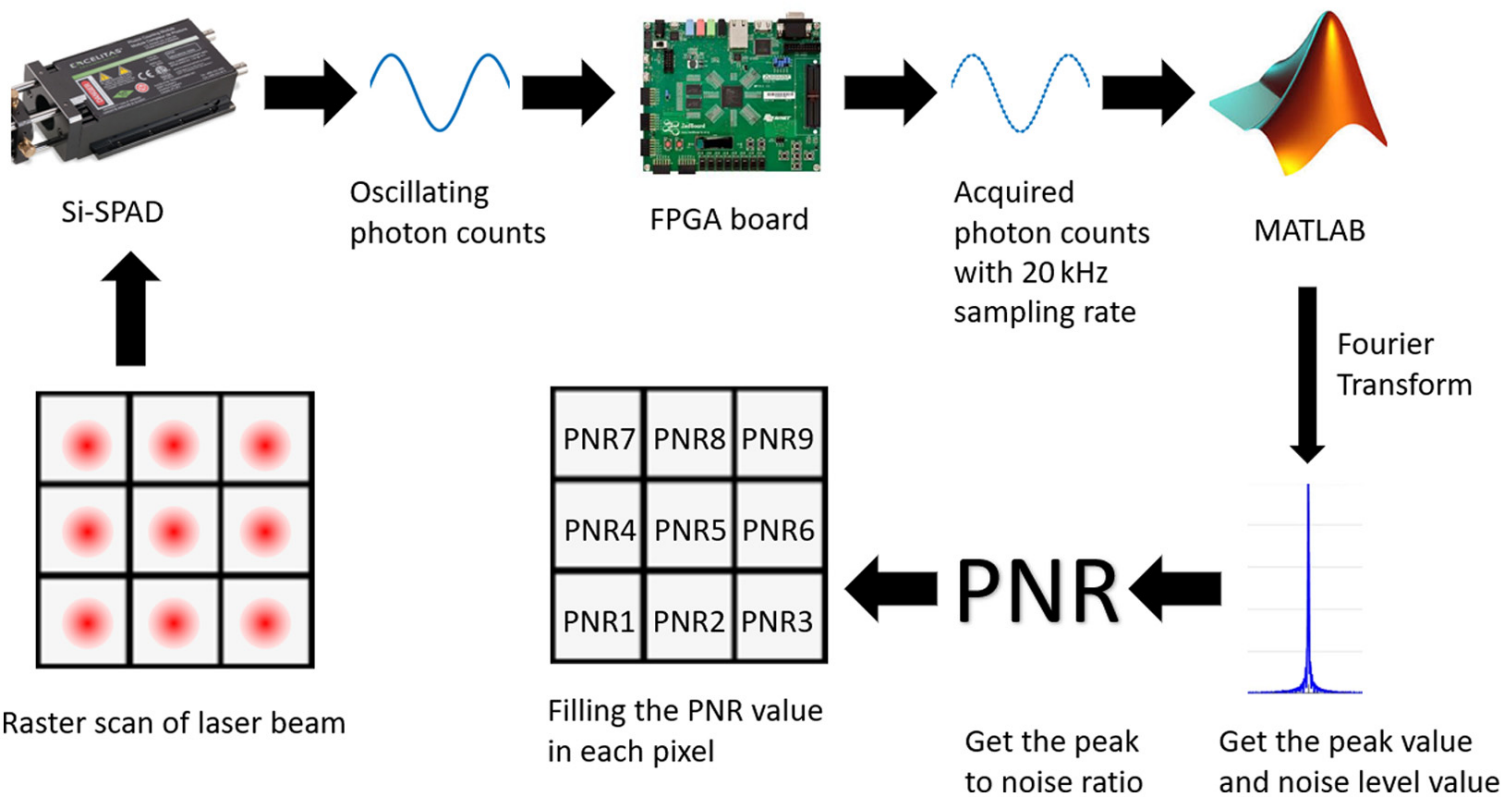
Flow chart of how PNR value is acquired for each pixel.

The reconstruction procedure for the elasticity-contrast images is illustrated in Fig. 3. With a sound wave at a fixed driving frequency, the lateral raster scan of the laser beam starts from the bottom left and ends at the top right. For each pixel, the FPGA board takes the Si-SPAD output over a total of 1999 time bins. Each bin has a 0.05 ms dwelling time, thus having a 20 kHz sampling rate. This follows the Nyquist theorem, where the maximum resolvable frequency is half of the sampling rate. As the maximum frequency we send to the agar sample is 6900 Hz, the 10 kHz limit is adequate for the whole experiment. Each pixel has a different tilt angle as set by the MEMS mirror, with 36 pixels both in a row and a column. After 36 rows of detection events, the optical delay moves by 1 ps and the beam starts from the bottom left again. This process is repeated until a total of 10 ps delay is scanned. For every pixel, we find the spectral response by performing the Fast Fourier Transform of the photon counting time series and get the PNR at driving frequency. Here, PNR is defined as the ratio between the amplitude of the spectrum at the driving frequency and the baseline amplitude, where the latter is calculated by averaging the amplitude over a frequency span between 100 and 300 Hz above the driving frequency.

**Fig. 4:**
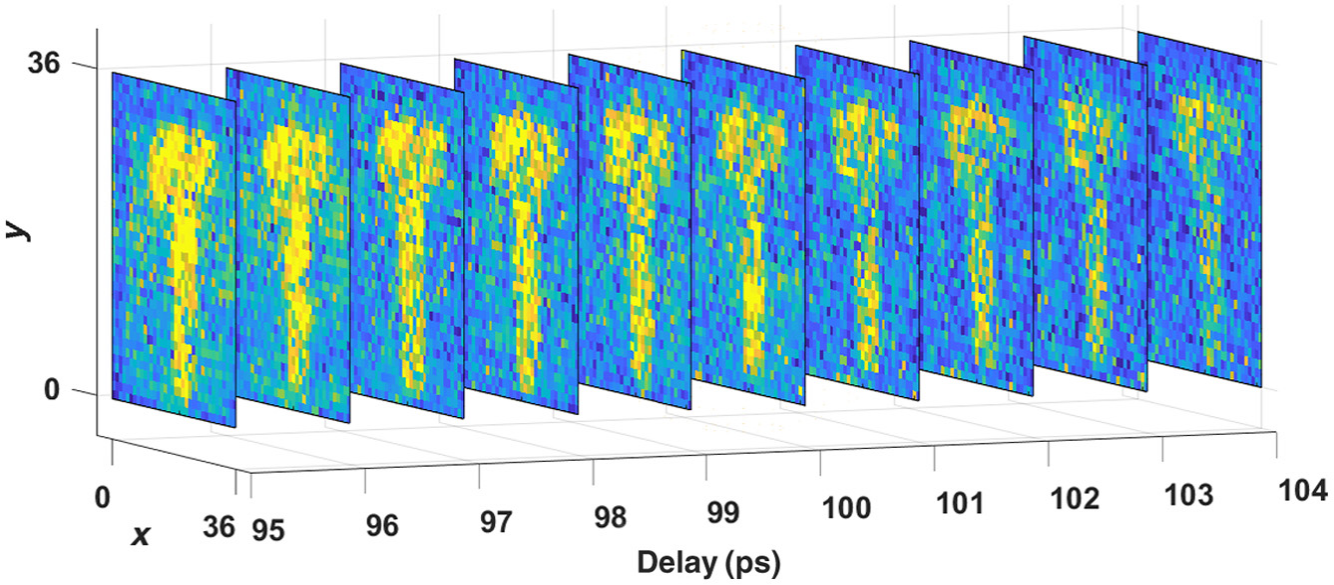
Display of 10 images get in one scanning. Each image is a reconstruction result with a delayline set to different picoseconds.

To form the elastogram, each pixel’s PNR is calculated for every delay setting. Hence, for each trial, we have a three-dimensional matrix with a total of 36×36×10 elements. Figure 4 shows an exemplar matrix obtained for a letter “T” agar sample with 2% W/W concentration. To get the best result, we only choose the maximum value along 10 ps. Finally, we have a 2D image with 36×36  pixels.

Besides conventional elastography, those delay scanning results in Fig. 4 may be further analyzed to form a “3D” elastogram, i.e., a layer-by-layer tomography of the samples’ elasticity. This is possible thanks to QPMS’ combined advantages in picosecond time gating, singe-photon counting, and multiscattering noise rejection,^11^ which implies new capabilities in sensing and imaging. A potential application is deep-tissue elastography, where the mechanical properties of an obscured target can be measured *in vivo*. Another is to map out the gradient stiffness of certain tissues, offering a detailed microphysical measurement. To that end, a deconvolution algorithm is warranted for data analysis because the photons backscattered from a point inside the tissue carry the frequency responses not only of that point but also from all layers above. This will be a subject of our future studies.

## 3 Results

**Fig. 5:**
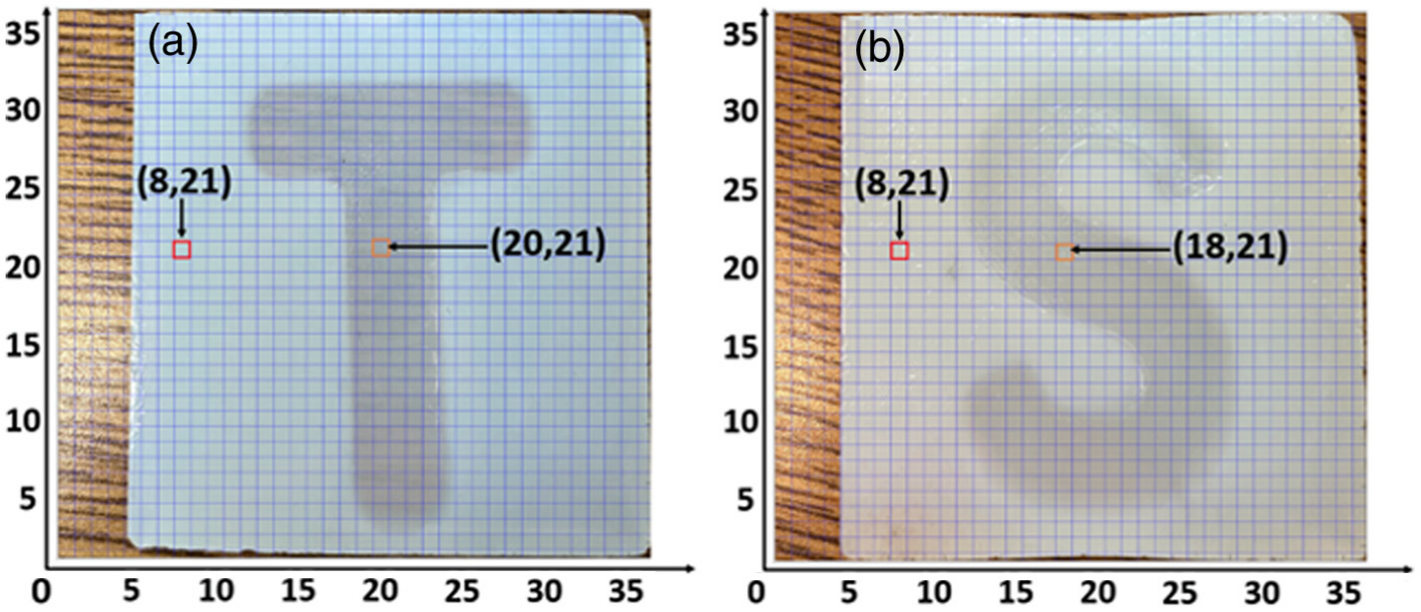
Photos of agar samples. Two squares indicate the position where we did the pretest, for each pixel the size is 0.15 cm width × 0.13 cm height. (a) Original photo of sample “T.” (b) Original photo of sample “S.”

We make two samples for this experiment, each containing a letter “T” or “S” made of 2% W/W agar and a frame made of 5% W/W agar. They have the same area dimensions of 4.85 cm by 4.85 cm but with a thickness of 0.80 cm for the “T” sample and 1.45 cm for the “S” sample. The “S” sample is thicker because of human error when making it. Figures 5(a) and 5(b) display their images from a front view, along with the MEMS mirror scanning grid (with the scanning step size of 0.15 cm along the horizontal and 0.13 cm along the vertical directions). Before the scanning, two pretests are conducted by directing the beam onto two points of different agar concentrations, as marked with squares in the figure. The pretest is performed by pointing the beam to a fixed pixel, detecting reflected photons, and stepping the sound waves from 600 to 6900 Hz with a 100 Hz interval. The total stepping time for all 64 frequencies is ∼15  s, so the photon counting integration time for each is ∼0.23  s. For each frequency, the sampling number of photon counts is rather constant, ∼4688. Note that the photon counting is not synchronized with the stepping. Instead, the oscillation amplitude of the received photon counts is obtained by performing a Fourier transform to the entire data set.

**Fig. 6:**
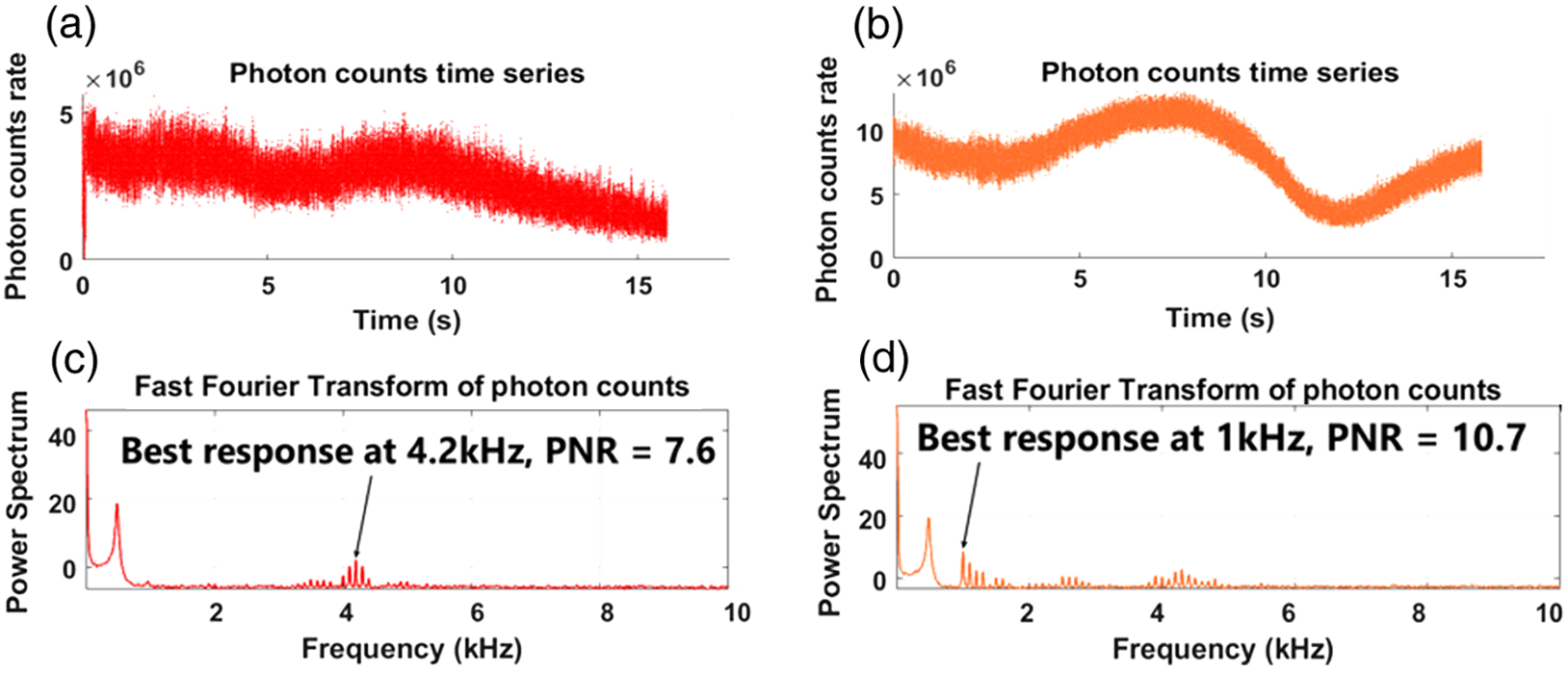
Pretest result for sample “T” with changing driving frequency. (a) Photon counts time series of pretest at pixel (8,21). (b) Same with (a) but at pixel (20,21). (c) Fourier transform result of panel (a). (d) Fourier transform result of panel (b).

Figure 6 shows the first pretest results for the “T” sample, where panels (a) and (b) plot the registered photon counts over 15 s for the 5% W/W and 2% W/W pixels in Fig. 5(a), respectively, as the sound frequency is swept. Panels (c) and (d) are their Fourier transformation results. In both figures, the high peaks at around 475 Hz arise from the vibration of the MEMS mirrors. For the 5% concentration, the response is significant only around 4.2 kHz. By contrast, for the 2% concentration, significant responses appear at around 1, 2.5, and 4.2 kHz, with the strongest at 1 kHz. This signifies the distinct Young’s modulus for different agar concentrations. That is, a higher concentration gives a larger modulus and thus a higher natural frequency.^25^ This suggests that, by selecting a specific frequency to drive the entire sample, one can observe differentiating frequency responses from agar samples of different concentrations. For the current samples, we choose 1 kHz as the driving frequency.

**Fig. 7:**
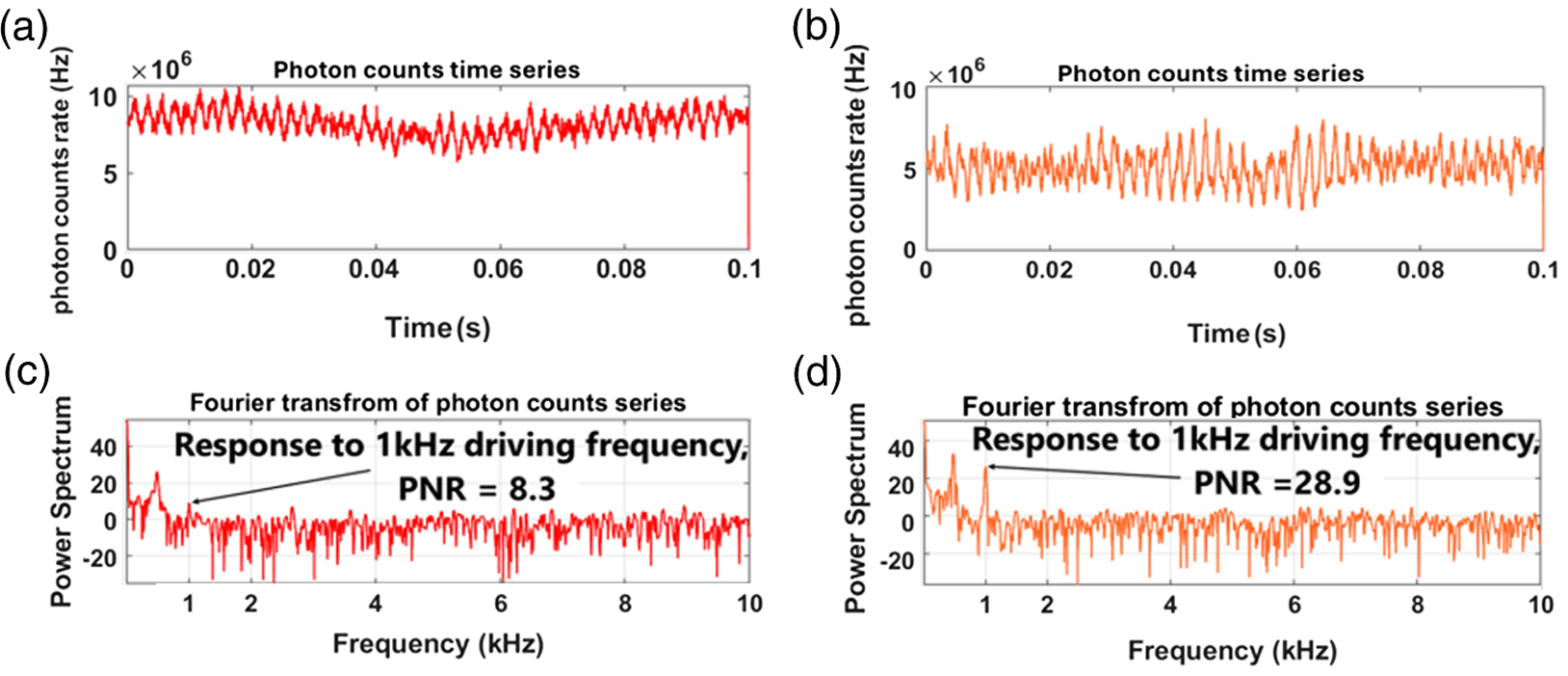
Pretest result for sample “T” with 1 kHz driving frequency. (a) Photon counts time series of pretest at pixel (8,21). (b) Same with panel (a) but at pixel (20,21). (c) Fourier transform result of panel (a). (d) Fourier transform result of panel (b).

Figure 7 shows the second pretest result by displaying photon counting histograms [Figs. 7(a) and 7(b)] and their Fourier transforms [Figs. 7(c) and 7(d)] for the two pixels in Fig. 5(a) under 1 kHz driving frequency. As seen in Fig. 7(d), the response at the driving frequency is much stronger for the 2% concentration, with a 28.9 PNR, as opposed to an 8.3 PNR in the 5% case shown in Fig. 7(c). This offers an approach to elastography by scanning the sample and recording the PNR at the driving frequency.

**Fig. 8:**
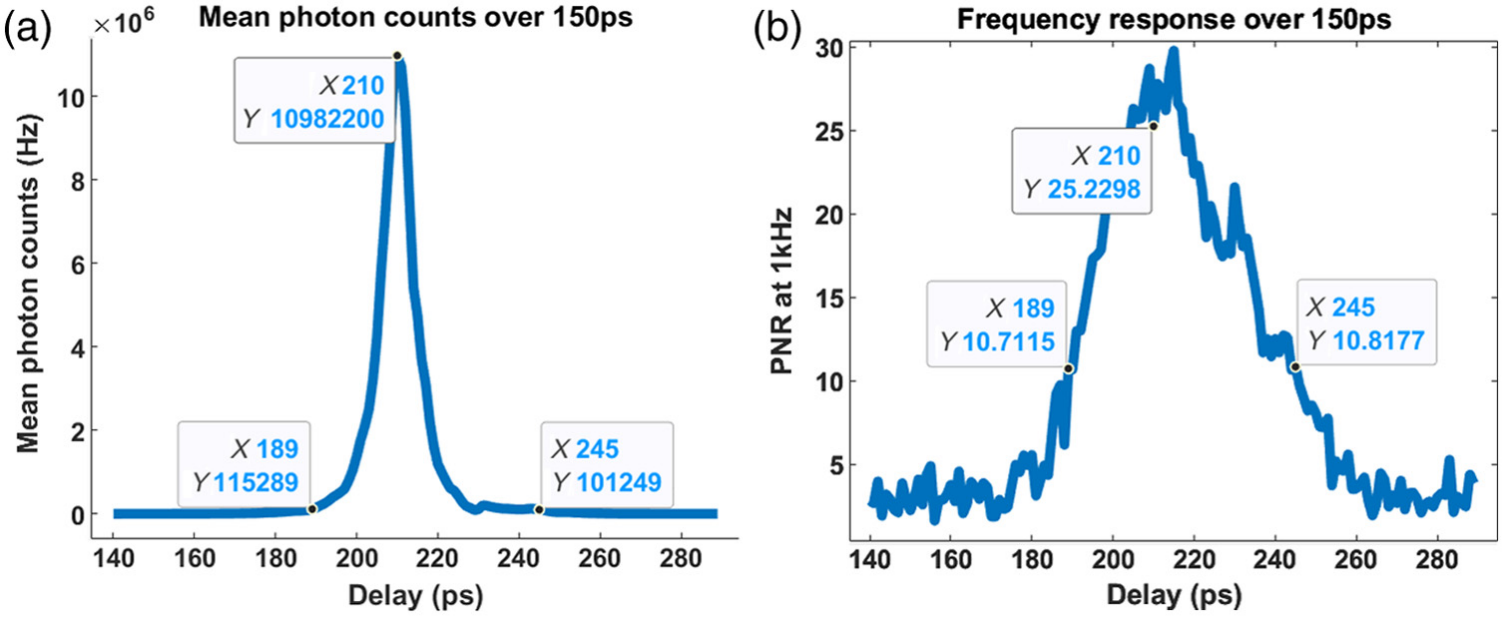
Delay scanning result for one pixel at 2% W/W agar along 150 ps. (a) Mean photon counts for each delay over 150 ps. (b) PNR at 1 kHz driving frequency for each delay over 150 ps.

To find the best scanning range for constructing an elastogram, we perform a delay scanning of the pump pulses at 1 kHz driving frequency for pixel (20, 21) in Fig. 5(a) over a range of 150 ps, corresponding to a 2.25 cm distance in the air to cover the whole thickness of the samples. The results are shown in Fig. 8, where the mean photon counts span over a 50 ps time window. The PNR, on the other hand, spans over a wider window, as PNR is defined as the ratio between signal and noise, whereby the absolute photon counts are normalized. With the help of this delay scanning result, we choose to move the delayline from 205 to 214 ps where we can get the best PNR.

**Fig. 9:**
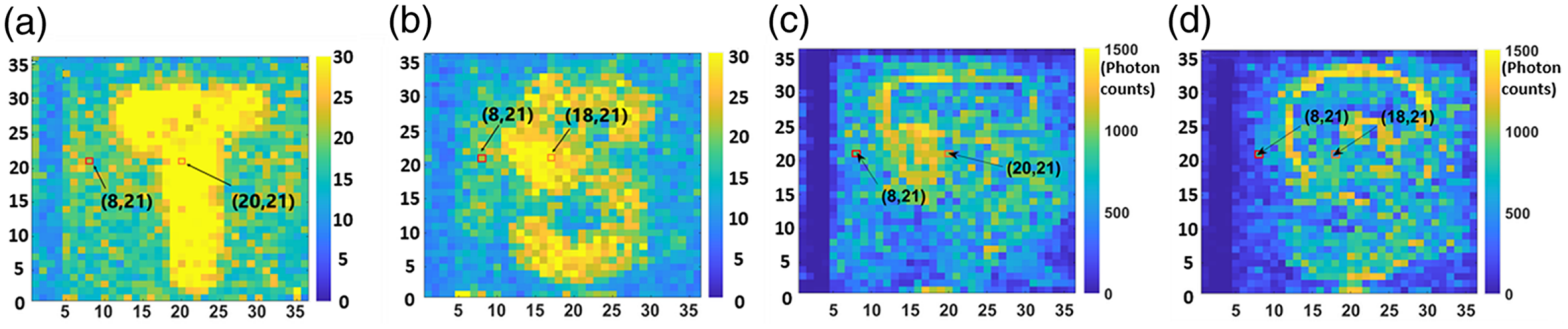
Image reconstruction results. Two arrows indicate the position where we did the pretest, for each pixel the size is 0.15 cm width × 0.13 cm height. (a) Sample “T” image reconstructed by plotting the PNR for each pixel. (b) Same with panel (a) but for sample “S.” (c) Sample “T” image reconstructed by photon counting only, with an average signal level of 478 photons per pixel per time bin. (d) Sample “S” image reconstructed by photon counting only, with an average photon count of 531 photons per pixel per time bin. In all figures, the left four columns do not cover the agar samples, as shown in Fig. 5.

Next, we perform the multi-pixel measurement for image reconstruction on two samples at 1 kHz driving frequency. The results are presented in Fig. 9, where panels (a) and (b) show the reconstructed images for the “T” and “S” samples, respectively. For each pixel, the size is 0.15 cm width × 0.13 cm height. As seen, the letters are in clear shapes, with distinct PNR values over the pixels of different concentrations. Comparing the two, the letter “T” has a more consistent high PNR than “S,” which might be caused by different acoustic wave propagation and excitation inside the two samples (the sound wave is applied from the left side of the samples). To verify that the reconstructed images in Fig. 9 indeed capture the samples’ Young’s modulus in different regimes and not their surface properties, we compare the PNR and direct photon count images. Figures 9(c) and 9(d) plot the highest mean photon counts as the pump is delayed for each pixel, respectively, for the samples “T” and “S.” As seen, in both cases, there is no clear difference in the received photon counting, as opposed to the PNR results.

**Fig. 10:**
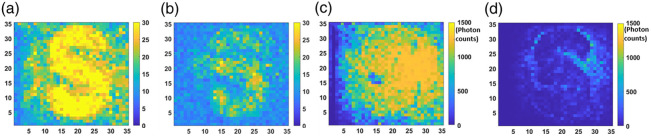
Reconstructed images of sample “S” under different illuminating laser power, for each pixel the size is 0.16 cm width × 0.14 cm height. Panels (a) and (b) plot the PNR values for each pixel with 836 and 84 mean counts per pixel per time bin, respectively. Panels (c) and (d) are their respective photon counting plots.

Last, we would like to note that the quality of the elastograms is dependent on the photon counts due to two reasons. One has to do with the detector noise, which comes from both the Si-SPAD dark counts and the Raman scattering in the QPMS waveguide. The other is due to the Poisson photon number fluctuation inherent in the signal. Both would require an adequate photon count to obtain appreciable PNR for each pixel. To study this effect, we repeat the experiment with a new sample (as agar samples cannot be stored for more than 3 weeks) and apply different illuminating signal power. Figures 10(a) and 10(c) show results for 0.84 mW signal power, and Figs. 10(b) and 10(d) are for for 0.16 mW. The mean photon counts per pixel per time bin in these two cases are 836 and 84, respectively. For each pixel, the size is 0.16 cm width × 0.14 cm height because the image size is 35×35. As seen, the reconstructed image is clear for the first case but becomes blurred as the mean photon count is reduced. This result is consistent with Ref. 10. In both cases, the PNR plots clearly resemble the concentration but not the surface reflectivity, which confirms the results in Fig. 9.

## 4 Discussion

In this work, we have successfully applied SPE to reconstruct elasticity-contrast images of 2% W/W agar letters embedded in 5% W/W agar blocks. While the results are promising, this study is only the first proof of principle, with more to follow. First, we have only tested agar samples of a single layer. As most tissues have multiple layers with disparate elasticity, another study is to test its performance in real biomedical soft tissues.

Second, we have yet to quantitatively measure the samples’ Young’s modulus. The next study is to find the relationship between the PNR value and Young’s modulus. For such, the frequency and intensity of the applied acoustic waves must be carefully accounted for. Also, pure water shall be used, and the samples shall be cooled slowly to reduce air bubbles, for higher-quality samples. While those imperfections are not important in the current contrast imaging experiment, they may lead to errors in correlating PNR to Young’s modulus.

Another area of improvement is the driving sound source. In this experiment, the intensity of the sound wave is not uniform across the frequency range, with the sound wave amplitude varying by up to 60 dB. Moreover, the stepping of the sound wave frequency is not synchronized with the data acquisition, so the amount of data points for each frequency is slightly different. While these do not affect the contrast imaging in this study (as the frequency stepping is only applied during the pretest), they may cause inaccuracy in quantitative measurements of Young’s modulus.

For our final result, both figures of the letter boundaries are somewhat blurred, showing a limited spatial resolution. This is because the laser beam size on the samples is 2 mm in diameter larger than the scanning grid size (0.15 cm width × 0.13 cm height). Also, the vibration of one pixel affects the neighboring pixels so that there is no sharp contrast between contacting 2% W/W and 5% W/W agar. To improve the resolution, one may focus the laser beam or use a smaller collimated beam.

## 5 Conclusion

In this study, we report a novel imaging modality based on optical elastography with single-photon counting, which has the advantages of non-contact, non-interferometric, long working distance, low-intensity optical illumination, and mode-selective single-photon detection. We demonstrate a proof-of-principal spatial mapping of agar phantoms (concentrations of 2% W/W and 5% W/W) at different elasticities. The spatial mapping is achieved under very low illuminating signal power at 0.16 mW resulting in 84 mean photon detections per pixel per time bin. This may allow *in vivo* measurement of vulnerable or light-sensitive tissues, such as cornea. Finally, our system is compact and in an all-fiber configuration, which is superior in size, weight, and robustness. The fundamental and practical advantages combined highlight a new approach to optical elastography.

## Data Availability

Data supporting the findings of the article are not publicly available at this time but can be obtained from the authors upon reasonable request.
